# Challenges of Assessing Community Mortality Due to Respiratory Viruses in Children Aged Less Than 5 Years

**DOI:** 10.1093/cid/ciab487

**Published:** 2021-09-02

**Authors:** Mauricio T Caballero, Ashish Satav, Christopher J Gill, Saad B Omer, Rachel C Pieciak, Abdul Momin Kazi, Eric Af Simões, Fernando P Polack

**Affiliations:** 1 Fundacion INFANT, Buenos Aires, Argentina; 2 Consejo Nacional de Investigaciones Científicas y Técnicas, (CONICET), Buenos Aires, Argentina; 3 Meditation, AIDS, Health, Addiction & Nutrition (MAHAN) (MAHAN) Trust, Mahatma Gandhi Tribal Hospital Karmagram, Utavali, Tahsil, Dharni, Amravati, India; 4 Department of Global Health, Boston University School of Public Health, Boston, Massachusetts, USA; 5 Yale Institute for Global Health, Yale University, New Haven, Connecticut, USA; 6 Section of Infectious Diseases, Department of Medicine, Yale School of Medicine, Yale University, New Haven, Connecticut, USA; 7 Department of Epidemiology of Microbial Diseases, Yale School of Public Health, Yale University, New Haven, Connecticut, USA; 8 Yale School of Nursing, Yale University, New Haven, Connecticut, USA; 9 Department of Pediatrics, The Aga Khan University, Karachi, Pakistan; 10 Department of Pediatrics, Section of Infectious Diseases, University of Colorado School of Medicine, and Children’s Hospital Colorado, Aurora, Colorado, USA; 11 Department of Epidemiology, Center for Global Health Colorado School of Public Health, Aurora, Colorado, USA

**Keywords:** community mortality, challenges, respiratory syncytial virus

## Abstract

**Background:**

Estimating the real impact of respiratory syncytial virus (RSV) disease is key for the development of vaccines and treatments. Ascertaining the burden of community mortality due to RSV is challenging due to the lack of primary data. Therefore, conducting observational studies to determine the factors associated with community mortality due to the virus in developing countries is important.

**Objective:**

Our aim in this study was to describe the obstacles, gaps, and challenges that investigators face in low-income, vulnerable regions in 4 developing countries on 3 continents.

**Results:**

The main obstacles and challenges of ascertaining community mortality due to RSV were defining strategies to consent families for testing before burial, sampling individuals at the household level, supporting bereaved parents with different cultural and religious backgrounds, establishing tailored strategies for studies in challenging settings, and integrating RSV mortality data from nasopharyngeal samples.

**Conclusion:**

Detailed logistical planning based on population sociodemographic information, grief counseling, staff training, and a multidisciplinary approach with adequate laboratory infrastructure is critical to successful observational community-based RSV studies.

Lower respiratory tract infection (LRTI) due to respiratory syncytial virus (RSV) is the leading cause of hospitalization and an important cause of post-neonatal infant death worldwide [1, 2]. In 2015, RSV was estimated to cause approximately 3.3 million hospitalizations and 118 000 deaths [2]. However, estimating RSV deaths is challenging, particularly in cases that occur in communities where primary data are limited [3-5]. Moreover, RSV infection may serve as a precursor to other severe pulmonary illnesses during childhood in ways that are still poorly understood. For example, in a recent randomized, controlled trial that evaluated a candidate RSV vaccine for pregnant women to prevent LRTI in their offspring, a post hoc protective effect against all-cause pneumonia was observed through at least 6 months of age, suggesting that the burden of illnesses attributed to RSV may be underestimated [6].

Assessing the burden of RSV mortality is challenging for several reasons [3]. First, the majority of infant deaths occur in low- and low- to middle-income countries (LMICs) where molecular diagnostic methods are scarce [3]. Second, recent observations in minimally invasive tissue sampling (MITS) studies suggest that even when an agent is identified in the upper respiratory tract, understanding its role in the lungs during the fatal event is complicated [7]. Third, a significant proportion of infant deaths in LMICs occur in the community [1, 8]. Causes of community deaths are often tentatively attributed through verbal autopsies (VAs) to respiratory infections or sudden infant death syndrome (SIDS), but the sensitivity and specificity of these cause-of-death (COD) allocation methods are poor [4, 9]. Fourth, the findings described above from the maternal RSV vaccine study suggest that the impact of RSV in the community may exceed our initial speculations [6]. Fifth, attributing all fatalities caused by bronchiolitis syndrome to RSV may overestimate the pathogen’s burden and may inflate mortality rate estimations [2, 10-12]. Finally, complete diagnostic autopsies (CDAs) remain the gold standard for COD ascertainment. However, the limited availability of pathologists; the challenges in studying cases before the body is disposed of; and the numerous social, religious, and personal beliefs and feelings in families during these tragic events hamper performance of CDAs in these situations [3].

Our goal in this study was to outline challenges at representative sites in LMICs on different continents (Buenos Aires, Argentina; Melghat, India; Lusaka, Zambia; Karachi, Pakistan) and propose strategies to more accurately assess the impact of RSV as a COD in young children worldwide.

## ARGENTINA, BUENOS AIRES

### Background

**Figure 1.**
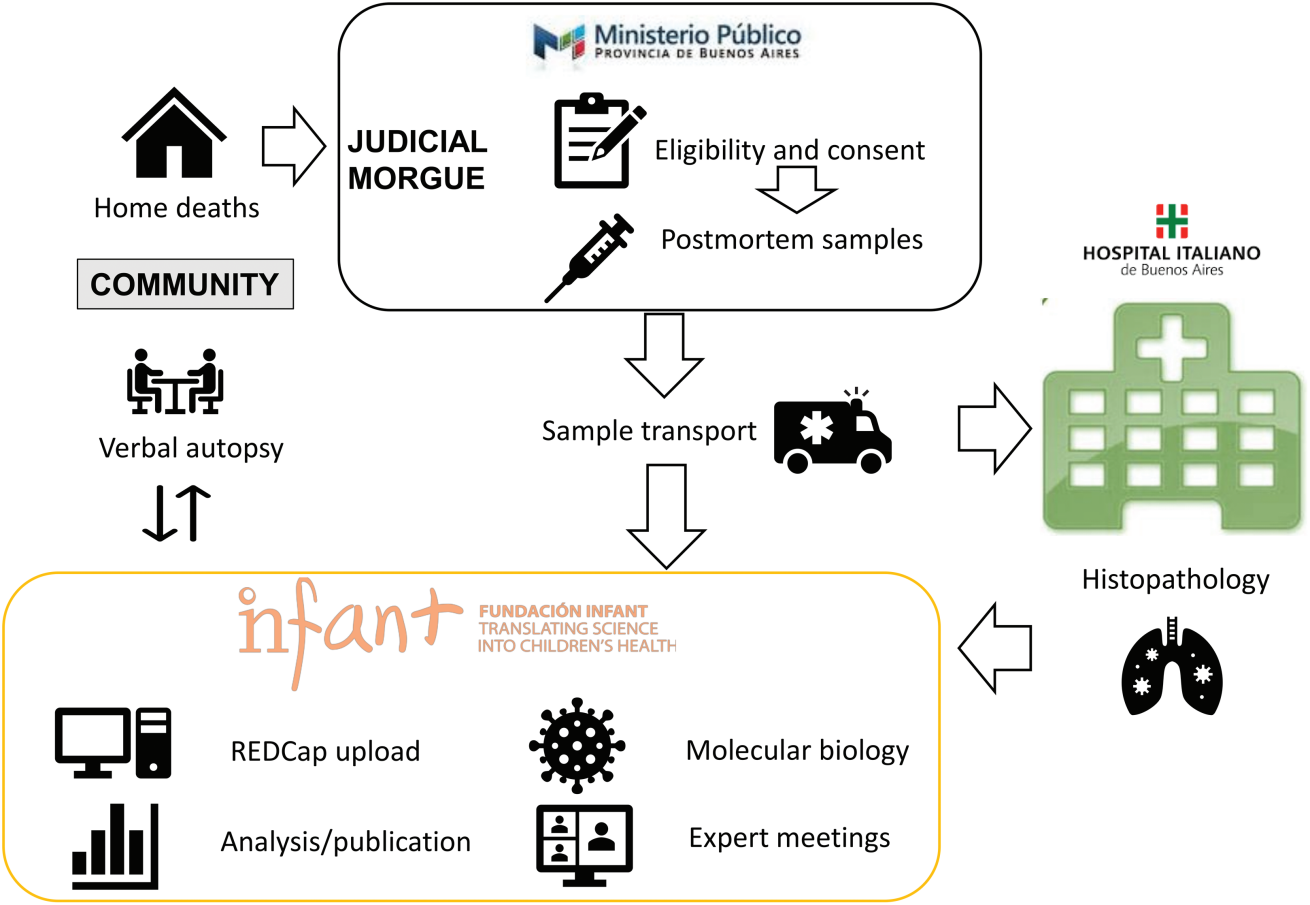
Argentina study road map. Abbreviation REDCap, Research Electronic Data Capture.

The southern region of Buenos Aires has a catchment population of 320 000 children aged <5 years (U5) living in 120 km^2^ (Table 1). There, exploratory studies to determine the role of RSV in community deaths in U5 children included an age-matched, case-control, population-based study using VAs (modified from the World Health Organization instrument). This was supplemented with a study of real-time polymerase chain reaction (RT-PCR) detection of viral RNA in nasal secretions of fatal lung disease at the local morgue and an ongoing evaluation of viral etiologies in fatal LRTI using viral RNA detection from nasopharyngeal swabs (NPS); MITS studies of histopathology combined with molecular detection of viral, bacterial, and fungal agents; and VAs followed by COD allocation using determination of COD (DeCoDe) diagnosis standards from the Child Health and Mortality Prevention Surveillance Network (Figure 1) [13, 14]. Using the DeCoDe method, a panel of experts analyzed all individual information and established the underlying and immediate COD based on the *International Classification of Diseases, 10th Revision*, and the World Health Organization death certificate [13, 14].

**Table 1.** Summary of General Characteristics of Each Study

Site	Enrollment Level	Catchment Population	Approach
Buenos Aires, Argentina	Community	320 000	MITS, NPS, VA, DeCoDe
Melghat, India	Community	13 000	NPS, MR, VA, MITS
Lusaka, Zambia	Community/hospital	300 000	NPS, MR, VA, MITS
Karachi, Pakistan	Community	250 000	NPS, VA, MITS

Abbreviations: DeCoDe, determination of cause of death standard; MITS, minimally invasive tissue sampling; MR, medical records; NPS, nasopharyngeal swab; VA, verbal autopsy.

### Results to Date

In our first study, we obtained samples from children U5 who died in the community and were sent to the morgue for full necropsy between May 2016 and April 2017. Then, we analyzed 114 nasopharyngeal samples using RT-PCR; 53 were positive for at least 1 virus (46.5%). From all positive samples, 11 had RSV (20.7%). The community mortality rate associated with RSV was 0.26/1000 live births (95% confidence interval [CI], .08–.45) [15]. Using parallel in-hospital mortality studies in the same region, we estimated that for every child who died at the hospital, another child died in the community (in-hospital mortality rate, 0.28/1000 live births) [11]. Risk factors for community death included incomplete vaccination (odds ratio [OR], 3.39; 95% CI, 1.20–9.62), no running water at home (OR, 4.39; 95% CI, 1.11–17.38), crowding (OR, 3.73; 95% CI, 1.41–9.88), having an adolescent mother (OR, 4.89; 95% CI, 1.37–17.38), admission to a neonatal intensive care unit (OR, 7.17; 95% CI, 2.21–23.27), and lack of primary care/emergency department visit during a fatal event (OR, 72.32; 95% CI, 4.82–1085.6) [15].

### Political Challenges

In Argentina, deaths that occur outside of healthcare facilities are legally considered to be of “uncertain cause”; therefore, the Justice Department is mandated to request forensic necropsies. Yet, the Buenos Aires Justice Department lacks financial and logistical resources to complete a thorough COD assessment. Thus, most infant deaths in the community that lack overt signs of violence are labeled as SIDS. Therefore, the rate of SIDS in community fatalities far exceeds international numbers, affecting public health policies that are mainly targeted to prevent this problem. To investigate community deaths in children U5, our Argentinean team collaborated with the state prosecutor’s office in Buenos Aires to access tissue samples at the local morgues. This collaborative effort presented communication challenges between the 2 teams conducting important work from very different perspectives: criminal investigation and COD ascertainment for public health purposes. Advancing the program demanded educating prosecutors about the magnitude of the public health problem (which had remained virtually unseen by the society), establishing a stepwise approach to competing priorities, and identifying synergies between both teams.

### Logistical Challenges

We trained and established a dedicated team at the morgue to consent families and conduct diagnostic procedures, preserve samples, and prepare samples for transportation to the laboratory. To prevent interference with routine practices, we integrated our regulatory team into the team at the morgue and laid out a meticulous logistical plan. We trained all participants in good clinical practices, established standard operating procedures, and controlled for quality of data and samples through designated team members and an external pathologist. Given that families needed guidance, we also advised families about routine procedures to recover the body and sort through unnecessary bureaucratic delays. In addition, we provided grief counseling interventions that continued through home visits and VA interviews. Previously trained psychologist, pediatricians, and social workers were responsible for counseling and VA interviews. Allocation of the final COD was performed using the DeCoDe method [13, 16].

### Cultural and Religious Challenges

Our study did not face significant cultural or religious challenges. The community suffers from 10% reported violence at home, 13% of families admitting that a member has drug abuse problems, and 57% of adults formally unemployed. More than 90% of parents consented to participate in the study.

## INDIA, MELGHAT

### Background

Melghat is a hilly, forested, difficult-to-access tribal area located in the northern part of the Amravati District of Maharashtra State in India. The population is approximately 300 000 with 75% tribal communities (Korku is the major tribe). The tribal population live in small, overcrowded huts (made from mud or thatch) without electricity and in extreme poverty [17, 18]. Child mortality and malnutrition is high (U5 mortality rate is > 80/1000 live births), and deaths occur mostly in the community (>67%) as a result of the absence of primary healthcare centers in most of the villages [19]. Since September 2016, the Indian team has been conducting an observational study to estimate the burden of child mortality and pneumonia due to RSV infection at both the community and health facility levels. For this purpose, NPS were collected from children with LRTI and from those who died in 93 villages and 2 government hospitals, 1 charitable hospital (MAHAN), and 5 primary health centers in the region (Table 1) [19]. The NPS were tested for RSV and other respiratory viruses. In 2020, the sampling procedure was expanded to the MITS approach.

### Logistical Challenges

Access to Melghat is complicated, the scarcity of transport facilities, poor road conditions, heavy rains that isolate villages during the rainy seasons, and the lack of communication systems make the transportation of NPS from villages to the laboratories difficult. Furthermore, the irregular electricity supply hampers storage of the samples for prolonged periods. To solve these problems, samples were stored in PrimeStore Molecular Transport Media (MTM) viral transport medium, which stabilizes nucleic acids at room temperatures, in 4ºC refrigerators at the MAHAN hospital and sent to the National Institute of Virology Pune periodically for assay. Deaths that occurred outside the village, deaths that occurred late at night, funerals that were conducted in the early morning, and refusal by parents hampered sample collection.

### Cultural and Religious Challenges

More than 84% of the population belongs to different tribal communities with different dialects and beliefs. More than 60% of the population is illiterate or semi-illiterate. In this context, communication regarding study procedures was challenging. Deaths that occur during the night result in funerals that occur early the next morning or even during the night. However, news of nocturnal deaths reach the village healthcare workers who are local trained married semiliterate tribal females later, hampering NP sampling.

To overcome these challenges, we trained our village healthcare workers and supervisors, established procedures for tracing deaths in the study area, educated the population, and established a bond of trust to ensure the rapid reporting of deaths. In addition, we provided grief counseling to parents and families. We also provided free treatment to all children aged <5 years.

### Community Staff Challenges

An important challenge for our study was the enrollment of qualified field staff. Healthcare workers are reluctant to work in Melghat mainly due to the harsh terrain. Hence, we had to train local tribal and often semiliterate youth to conduct the study procedures in the villages, including collection of NP samples. In addition, in order to the collection of NP samples, we expanded our pool of trained workers to include those of the integrated child development schemes, accredited social health activists, auxiliary nurse midwifes, traditional birth attendants, and traditional faith healers. We also used our trained local tribal youth counselors at all study hospitals for collection of NPS, grief counselling, and other study procedures.

## ZAMBIA, LUSAKA

### Background

The Zambia Pertussis/RSV Infant Mortality Estimation (ZPRIME) study was a 3-year, prospective, community-based, post-mortem, prevalence study designed to identify the proportion of infant deaths in Lusaka, Zambia, that are attributable to *Bordetella pertussis* and RSV. In Lusaka, nearly all infants who die are seen at the University Teaching Hospital (UTH). There, a death certificate (with a COD) is issued to the family. By law, a deceased infant cannot be buried without issue of a burial permit, which can only be obtained once a death certificate has been issued. Since these are primarily issued at UTH, we were reassured that the burden of community-acquired deaths in Lusaka is accurately captured (89% of cumulative consent rate).

**Figure 2.**
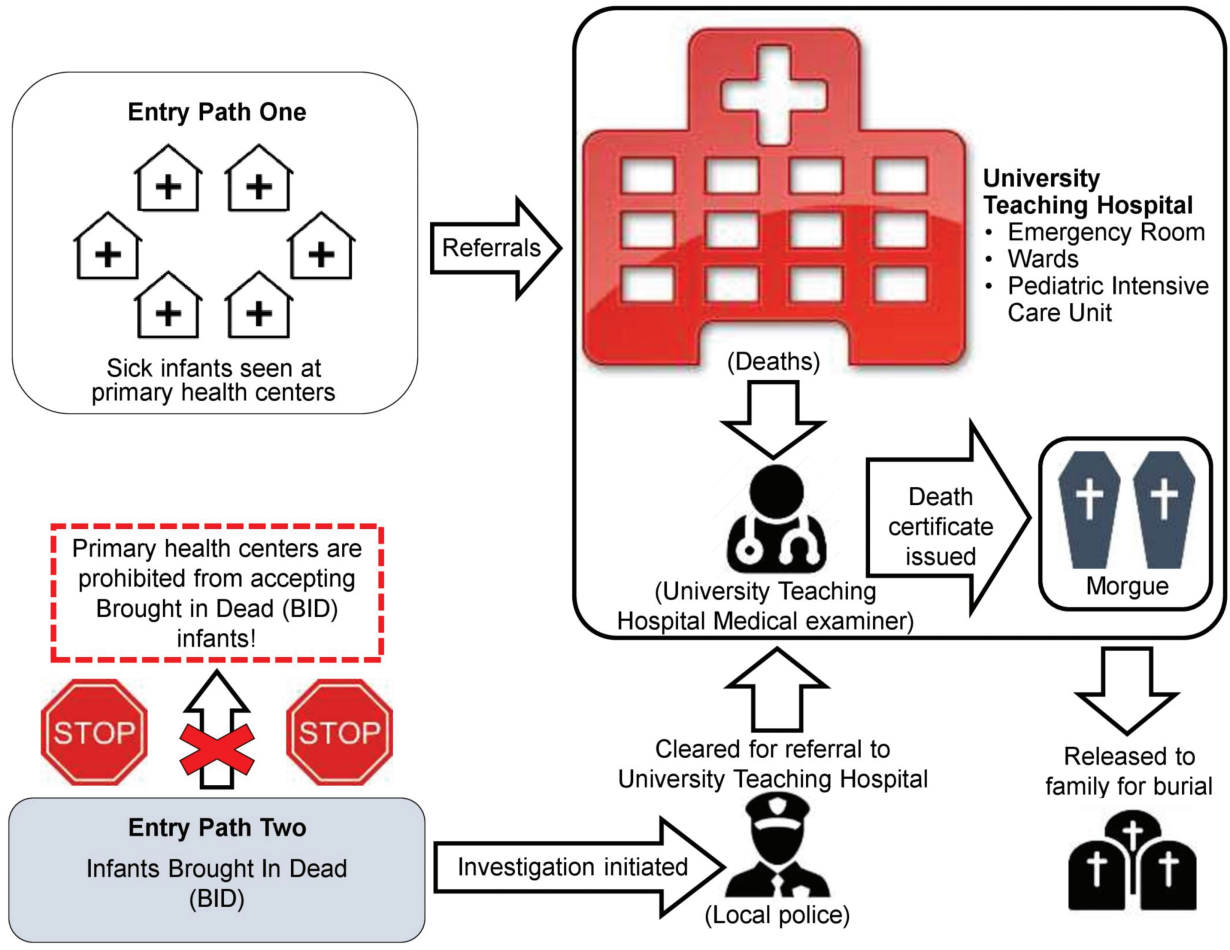
Flow chart of the Zambia Pertussis/RSV Infant Mortality Estimation enrollment strategy.

Infants (aged 4 days to <6 months) are identified via 2 pathways (Figure 2). The first entry path consists of all infants who died at UTH or were referred there from healthcare facilities within the city (Table 1). The second entry path consists of infants who are “brought in dead” (BID) to UTH or to 1 of the 4 satellite hospitals. Infants who die in the community must first be evaluated by the local police to rule out criminal cause. Then, they are able to be seen by the medical examiner at UTH.

Upon an infant death notification, a study staff member approaches the infant’s next of kin/guardian and offers grief counselling. After obtaining the next of kin/guardian’s consent, counsellors collect a medical history, a VA (for any BID infant), and a nasopharyngeal sample. These VAs along with information extracted from clinical or medical examiner records are used to characterize the circumstances of the death. All deaths are categorized as “respiratory” or “nonrespiratory” based on the information available through the VA, clinical records, and medical examiner reports. This system is indirect and relies on possibly biased information, which could result in an underestimation of RSV burden. To address this, a MITS was introduced in January 2020 to enroll 150 community infant deaths using a matched case-control design. The goals of the ZPRIME-MITS study are to calibrate the process for adjudicating infant deaths as respiratory vs nonrespiratory and to establish the concordance rate between PCR from NP samples to histological evidence of RSV-LRTI. With this new approach, we expect to improve the specificity of the RSV mortality adjudication process. Since 2020, the study has added MITS for those infants whose parents accepted the procedure.

### Results to Date

Between 31 August 2017 and 31 August 2020, ZPRIME enrolled 2286 deceased infants. Of these, 1412 (62%) were community deaths and 874 (38%) were facility deaths. RSV infections were prevalent in both settings, being identified among 112 of 1412 (7.9%) community and 50 of 874 (5.7%) facility deaths, respectively. When considering these totals as a function of total RSV deaths, the concentration in the community becomes more apparent, with 112 of 162 (69%) occurring in the community. Similarly, most RSV deaths occurred in infants aged 4 days–3months (82 of 112, 71.6%). These data confirm that RSV deaths largely occur in the community in younger infants and that, by extension, hospital-based surveillance alone will underestimate RSV mortality. In Zambia, RSV cases were most common in the spring of each year and geographically were concentrated in the poorest and most densely populated Lusaka city wards.

### Logistical Challenges

The success of ZPRIME hinges on the ability to enroll participants who are as representative of the population as possible. Thus, it was critically important for us to consider the potential challenges that were posed by obtaining informed consent. After several discussions with our local team members, scientists, medical anthropologists, and psychologists and with input from the local institutional review board (IRB), we developed the grief counseling protocol that we agreed to offer to families up-front regardless of their decision to consent to the study.

It was important to hire staff who would be able to distribute the workload and support each other through difficult conversations with bereaved relatives. Moreover, we partnered with a local psychiatrist to offer services and support for the team and individual team members on a regular basis. We also provided a quarterly group activity to provide the team with some relief from the stress of daily counseling with bereaved parents. Last, we encouraged our team members to take vacation leave as needed.

### Cultural and Religious Challenges

In our study, we did not face any significant cultural or religious challenges. However, our method for offering grief counseling to bereaved guardians as an unconditional incentive did provide insight into grief and grieving in this context. Since UTH is the primary site for issuing death certificates (which are required to inter a body), UTH is often the last stop for an infant’s guardians/next of kin before they are able to bury their child. So, when our study staff encountered these individuals at the morgue, they were often under tremendous time pressure, with only approximately 20 minutes for the grief counseling session. In that sense, these sessions probably did not achieve our goal of offering formal, structured grief counseling but rather became sessions more akin to trauma counselling, where the objective is to offer emotional support, empathy, and a chance for bereaved family members to express their sadness. After much discussion, our team attempted to offer more in-depth grief counseling sessions. However, very few families used this opportunity, which led us to discontinue the effort. We speculated that the causes for this disinterest and consulted several of our Zambian colleagues who offered insights into their own grieving practices. Open questions that we consider essential are whether this approach was seen as “too Western” to be culturally acceptable in Zambia and whether the families felt that traditional methods for grieving were preferable and/or sufficient. Since so few clients returned, we cannot know the reason why this approach failed to gain traction. In the future, if we were to offer additional support services to these families, we can take the lessons learned from this experience to inform future work in this area.

## PAKISTAN, KARACHI

### Background

**Figure 3.**
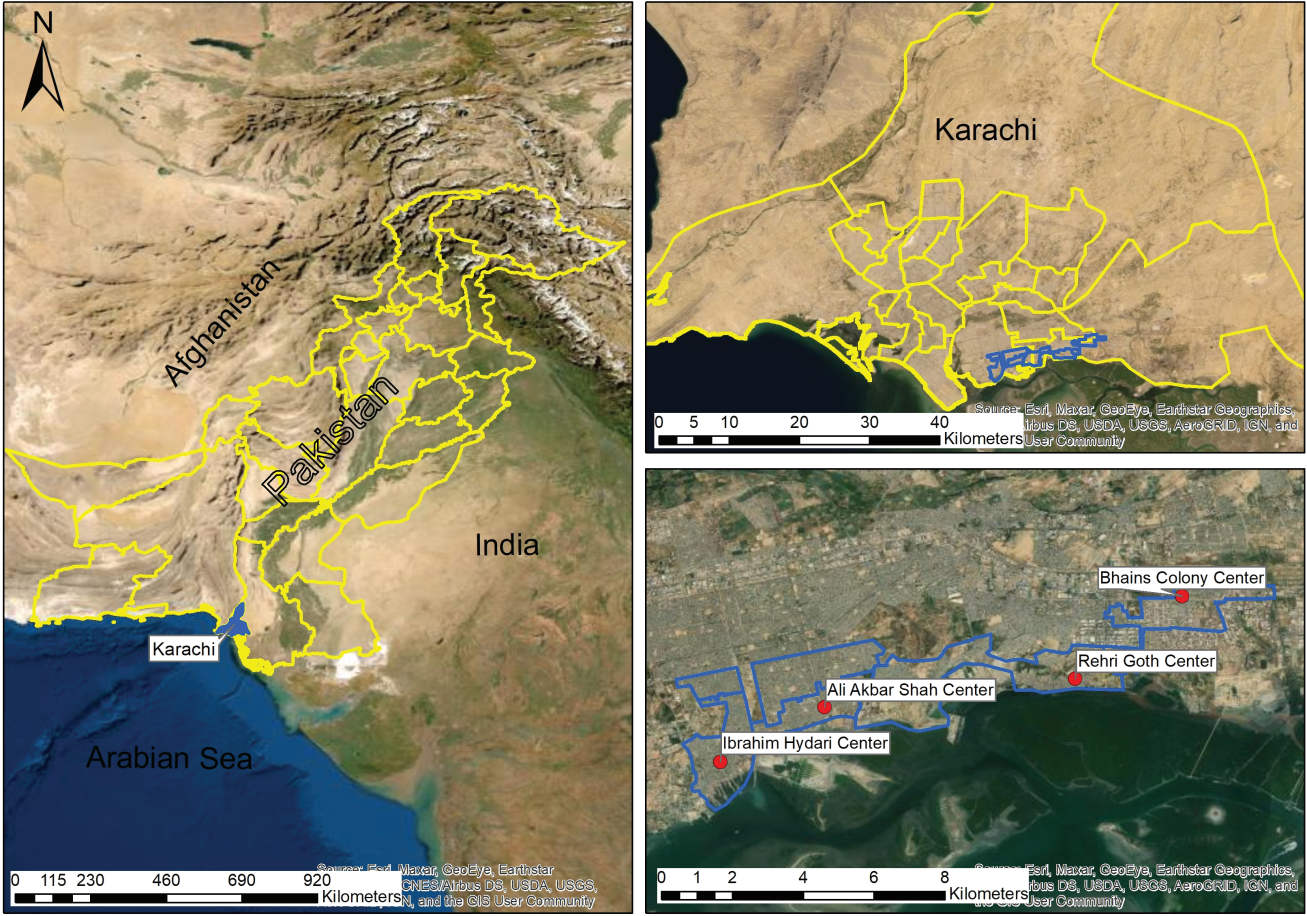
Map of Pakistan study catchment areas.

We set up an RSV mortality community-based surveillance from August 2018 to December 2020 in 4 peri-urban seaside settlements of Karachi, Pakistan (Figure 3), that consisted of a religiously conservative, low-income population (Figure 2). A health and demographic surveillance system was established in these areas to run primary healthcare centers (PHCs). Surveillance is conducted on married women of reproductive age and their children aged <5 years. Currently, information is updated every 2 months by community healthcare workers. The overall population of the catchment area is approximately 250 000 with a cohort of 8264 pregnancies and 7525 new births per year (Table 1) [20].

### Results to Date

A formative phase was conducted to identify standard operating procedure (SOPs) for the study’s surveillance phase. From interviews and focus group discussions with key stakeholders, it was evident that the community required education regarding RSV and the nasopharyngeal swab procedure, focusing on religious and sociocultural norms. Religious rulings regarding the permissibility of the procedure on a deceased infant for medical research purpose were obtained from Muslim, Hindu, and Christian scholars and advocated for in the community by local religious leaders. Methods of community engagement were identified, such as circulating pamphlets at the household level, mosque/church announcements, sessions with community elders and leaders, medical camps for neighborhoods, and community meetings. In addition, it was determined that grieving families would prefer to speak to a person from their own community and that a COD report or laboratory findings would be an appropriate incentive for parents.

Between August 2018 and December 2019, 927 eligible deaths occurred; 397 nasopharyngeal specimens were collected, and 13 tested positive for RSV preliminarily. The enrollment criteria consisted of stillborn or deceased infants (aged 0 days to 6 months), born and deceased in the catchment areas. In November 2020, MITS specimen collection was added to the program.

### Logistical Challenges

With a short time window for enrollment (due to religious beliefs regarding quick burial), study vans were available at all sites ready to mobilize the staff. Mobilizing at night for nocturnal death alerts was a particular challenge, mitigated with shifts for health workers. With this system, the team was able to approach a household within 25 minutes, on average, of a death alert. Death alerts were reported by key community partners (graveyard personnel, funeral personnel, traditional birthing attendants, healthcare workers, hospital and clinic staff, religious leaders, community leaders). Social mapping was conducted to identify routes and to maintain liaison with community partners who would give alerts. A real-time dashboard hosted on a secure cloud server was used to monitor study procedures.

### Cultural and Religious Challenges

Families were approached in a timely manner before infants’ bathing/burial for the consent process. This is a very sensitive time, and we used strategies such as involving key community partners to advocate for the study and staff training in communication and counseling skills. The family approach team was comprised of community mobilizers and healthcare workers who resided in the same community/neighborhood. Continued community engagement strategies were used to advocate for the study and obtain parental consent in the appropriate timeframe. Post-death services such as grief counseling with a psychologist for complicated grief cases, grief support home visits, and a COD consultation by a physician were offered to enrolled parents. These services were found to be an appropriate incentive for enrolling parents and contributed to community engagement.

### Community Staff Challenges

The study team was trained in mental health first-aid skills, compassionate listening, communication skills, and grief counseling. Further, the team held weekly group sessions, monthly individual sessions, and annual retreats with the psychologist regarding their mental health and resilience. Because healthcare workers from other studies operating in the same community had concerns about community mortality research, most study activities were conducted outside the PHCs to avoid adverse effects/stigmatization. Staff were trained in verbal consent script, community sessions material, and the enrollment strategy focused on community advocacy rather than sick infant surveillance.

## Conclusions

Implementation of community-based studies with specimen collection in the developing world has offered a novel experience for better assessment of the burden of RSV mortality.

Feasibility and success of the studies depended on different strategic aspects. First, an accurate knowledge of the capture population with active participation in decisions by local leaders and stakeholders was crucial. Second, screening and enrollment strategies needed to be planned in detail. Third, approaching bereaved parents and relatives through grief counseling was key for success in all studies. Fourth, training staff members with specific skills to approach families and to maintain the community liaison was a strong strategy. Fifth, building a multidisciplinary team with field staff, community members, local leaders, physicians, researchers, social workers, and mental health experts that work collaboratively is also important. Finally, migrating from NP swab toward a MITS approach was a necessary and common path to achieve an accurate understanding of the role of RSV as a cause of LRTI mortality.

## Notes

***Supplement sponsorship.*** This supplement is sponsored by the Bill and Melinda Gates Foundation.

***Potential conflicts of interest.*** A. S. reports subcontract work with the University of Colorado, Denver, for the study. C. J. G. reports a grant from the Bill and Melinda Gates Foundation. R. C. P. reports a grant from the Bill and Melinda Gates Foundation. E. A. F. S. reports grants from the Bill and Melinda Gates Foundation during the conduct of the study; grants, personal fees, and nonfinancial travel support from Astra Zeneca Inc, Merck, Regeneron Inc, Roche Inc, and Pfizer Inc; personal fees, nonfinancial travel support, and Data and Safety Monitoring Board (DSMB) fees from AbbVie Inc; personal fees from Alere Inc; DSMB fees from GSK Inc; grants from Johnson and Johnson; and grants and nonfinancial support from Novavax Inc outside the submitted work. F. P. P. reports a grant from the Bill and Melinda Gates Foundation; grants and personal fees from Janssen and Novavax Inc; and personal fees from Bavarian Nordic, Pfizer, Sanofi, Regeneron, and Merck outside the submitted work. All remaining authors: No reported conflicts of interest to disclose. All authors have submitted the ICMJE Form for Disclosure of Potential Conflicts of Interest. Conflicts that the editors consider relevant to the content of the manuscript have been disclosed.
